# Seasonal movements and habitat use of African buffalo in Ruaha National Park, Tanzania

**DOI:** 10.1186/s12898-020-0274-4

**Published:** 2020-02-03

**Authors:** Annette Roug, Epaphras A. Muse, Deana L. Clifford, Randy Larsen, Goodluck Paul, Daniel Mathayo, Donald Mpanduji, Jonna A. K. Mazet, Rudovick Kazwala, Halima Kiwango, Woutrina Smith

**Affiliations:** 1 Karen C. Drayer Wildlife Health Center, University of California, 1089 Veterinary Medicine Drive, Davis, CA 95616 USA; 2 Utah Division of Wildlife Resources, 1594 West North Temple, Suite 2110, Salt Lake City, UT 84116 USA; 3 Ruaha National Park, Tanzania National Parks, PO Box 369, Iringa, Tanzania; 4 California Department of Fish and Wildlife, 1701 Nimbus Road Suite D, Rancho Cordova, CA 95670 USA; 5 Department of Plant and Wildlife Sciences, College of Life Sciences, Brigham Young University, Provo, UT 84602 USA; 6 Department of Veterinary Medicine and Public Health, Sokoine University of Agriculture, PO Box 3021, Morogoro, Tanzania; 7 Department of Veterinary Surgery and Theriogenology, Sokoine University of Agriculture, PO Box 3021, Morogoro, Tanzania

**Keywords:** African buffalo, Habitat use, Home range, Ruaha National Park, Tanzania

## Abstract

**Background:**

Assessing wildlife movements and habitat use is important for species conservation and management and can be informative for understanding population dynamics. The African buffalo (*Syncerus caffer*) population of Ruaha National Park, Tanzania has been declining, and little was known about the movement, habitat selection, and space use of the population, which is important for understanding possible reasons behind the decline. A total of 12 African buffalo cows from four different herds were collared with satellite transmitters. Movements were assessed over 2 years from 11 animals.

**Results:**

The space use of the individual collared buffaloes as an approximation of the 95% home range size estimated using Brownian bridge models, ranged from 73 to 601 km^2^. The estimated home ranges were larger in the wet season than in the dry season. With the exception of one buffalo all collared animals completed a wet season migration of varying distances. A consistent pattern of seasonal movement was observed with one herd, whereas the other herds did not behave the same way in the two wet seasons that they were tracked. Herd splitting and herd switching occurred on multiple occasions. Buffaloes strongly associated with habitats near the Great Ruaha River in the dry season and had little association to permanent water sources in the wet season. Daily movements averaged 4.6 km (standard deviation, SD = 2.6 km), with the longest distances traveled during November (mean 6.9 km, SD = 3.6 km) at the end of the dry season and beginning of the wet season. The shortest daily distances traveled occurred in the wet season in April–June (mean 3.6 km, SD = 1.6–1.8 km).

**Conclusion:**

The Great Ruaha River has experienced significant drying in the last decades due to water diversions upstream, which likely has reduced the suitable range for buffaloes. The loss of dry season habitat due to water scarcity has likely contributed to the population decline of the Ruaha buffaloes.

## Background

Understanding wildlife movements and habitat use is critical for species conservation and management on a landscape scale [1]. Information on emigration and immigration, habitat preferences, and herd interactions may be important for evaluating population dynamics [2]. Movement data can also be used to identify critical interfaces for potential disease transmission between wildlife and domestic animal species [3]. The movements and habitat preferences of large ungulate species such as African buffaloes (*Syncerus caffer*) has been studied in multiple locations across Africa, including South Africa [4], Botswana [5, 6], and Namibia [7, 8]. Migratory behavior ranged from resident [5], partially migratory [7], to long distance [8]. In Tanzania, African buffalo ecology has been studied extensively in the Serengeti in northern Tanzania [9], but limited information is available from other areas of Tanzania.

Over recent decades, the African buffalo population in Ruaha National Park in south-central Tanzania appeared to be declining. Subjectively, park staff and tourist guides reported seeing fewer buffaloes than in the past, and a decline in absolute numbers was confirmed when comparing aerial counts conducted in comparable areas in the dry seasons of 2004 and 2013 (https://www.haliproject.org, unpublished). In addition to the suspected decline in numbers, park staff and tour operators reported rarely seeing buffaloes in the wet season between November and May, but no information existed on annual herd movements and habitat use. Buffaloes are seasonally hunted in game reserves surrounding the park, but as herd movements were not known, it was unclear whether the park’s buffaloes constituted a part of the hunted population.

As an additional concern, Ruaha National Park’s main water source, the Great Ruaha River, has experienced significant drying since the 1990s due to water diversions for agricultural irrigation upstream [10]. Buffaloes are water dependent [11], and access to water determines which areas can be utilized by buffaloes in the dry season [6, 12]. Further, the reduction in flow of the Great Ruaha River has seasonally increased the pressure on remaining water sources, and possibly increased the interaction between livestock and wildlife at the park’s boundaries [13]. Local cattle herds are known to be affected by bovine tuberculosis and brucellosis, and bovine tuberculosis has been detected in 8 wildlife species outside the park [14, 15]. African buffaloes are considered maintenance hosts for bovine tuberculosis [16], and while this disease is not thought to cause population declines in buffaloes, it may increase the populations susceptibility to other stressors such as drought [17].

The Health for Animals and Livelihood Improvement (HALI) project, a University of California, Davis and Sokoine University of Agriculture, Morogoro collaborative project, and Ruaha National Park, partnered to investigate the apparent buffalo decline. Components of the study included an aerial population count, demographic surveys, health assessments, and marking buffaloes with GPS collars.

The GPS collars were used to determine movements as well as habitat use of African buffaloes in Ruaha National Park in order to better understand possible seasonal population impacts from herds entering surrounding reserves or risks of contracting disease from local livestock populations at the borders of the park. We expected that the main herds of the park completed seasonal movements, but mainly stayed within park boundaries while utilizing the wildlife management area near the Great Ruaha River and bordering village lands. Gathered information could contribute to understanding possible reasons for the observed population decline and benefit Ruaha National Park for future management of this species.

## Results

### Collaring

**Fig. 1:**
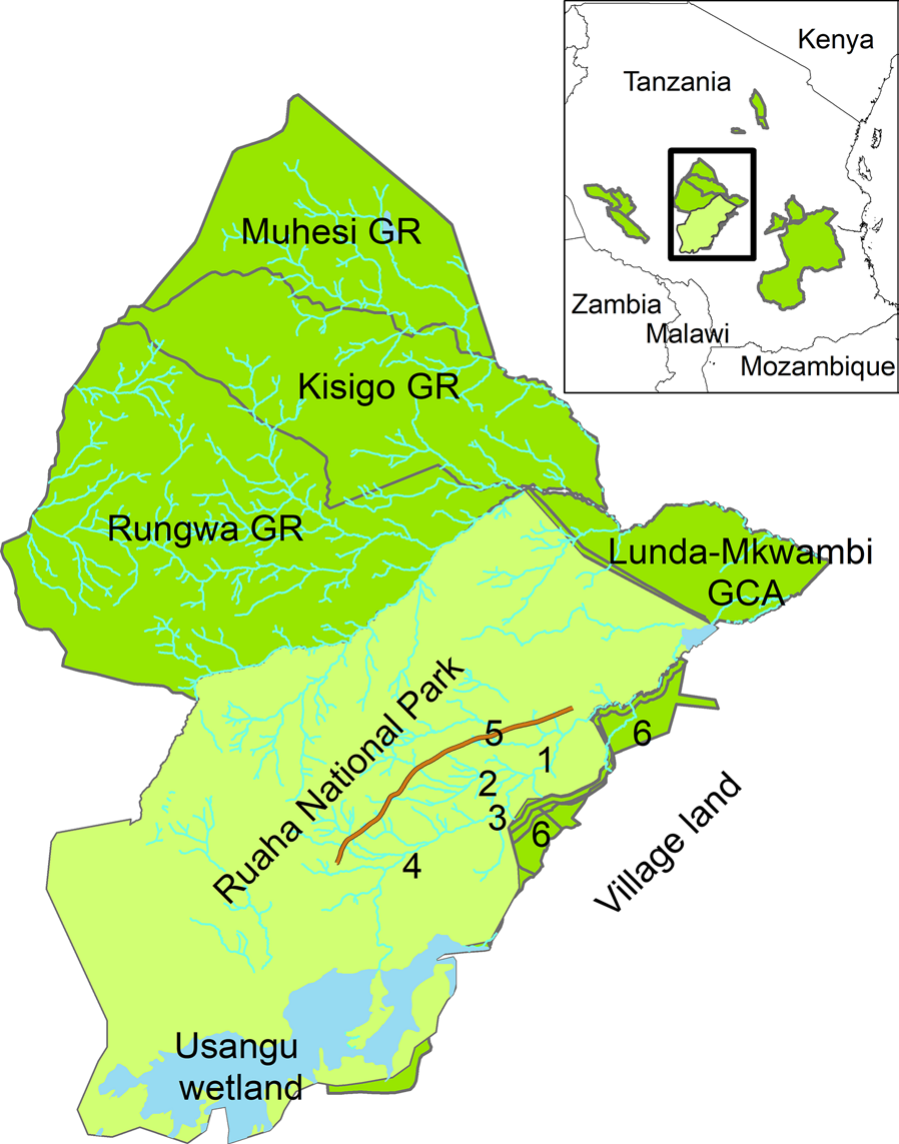
Overview of the Greater Ruaha Ecosystem in Tanzania and approximate capture locations of the collared African buffalo. 1 = Mwagusi, 2 = Mdonya, 3 = TelekiMboga, 4 = Jongomero. Other features shown: 5 = Escarpment, 6 = Pawaga-Idodi Wildlife Management Area. *GR* game reserve, *GCA* game controlled area (Creator and copyright holder: A. Roug)

**Fig. 2:**
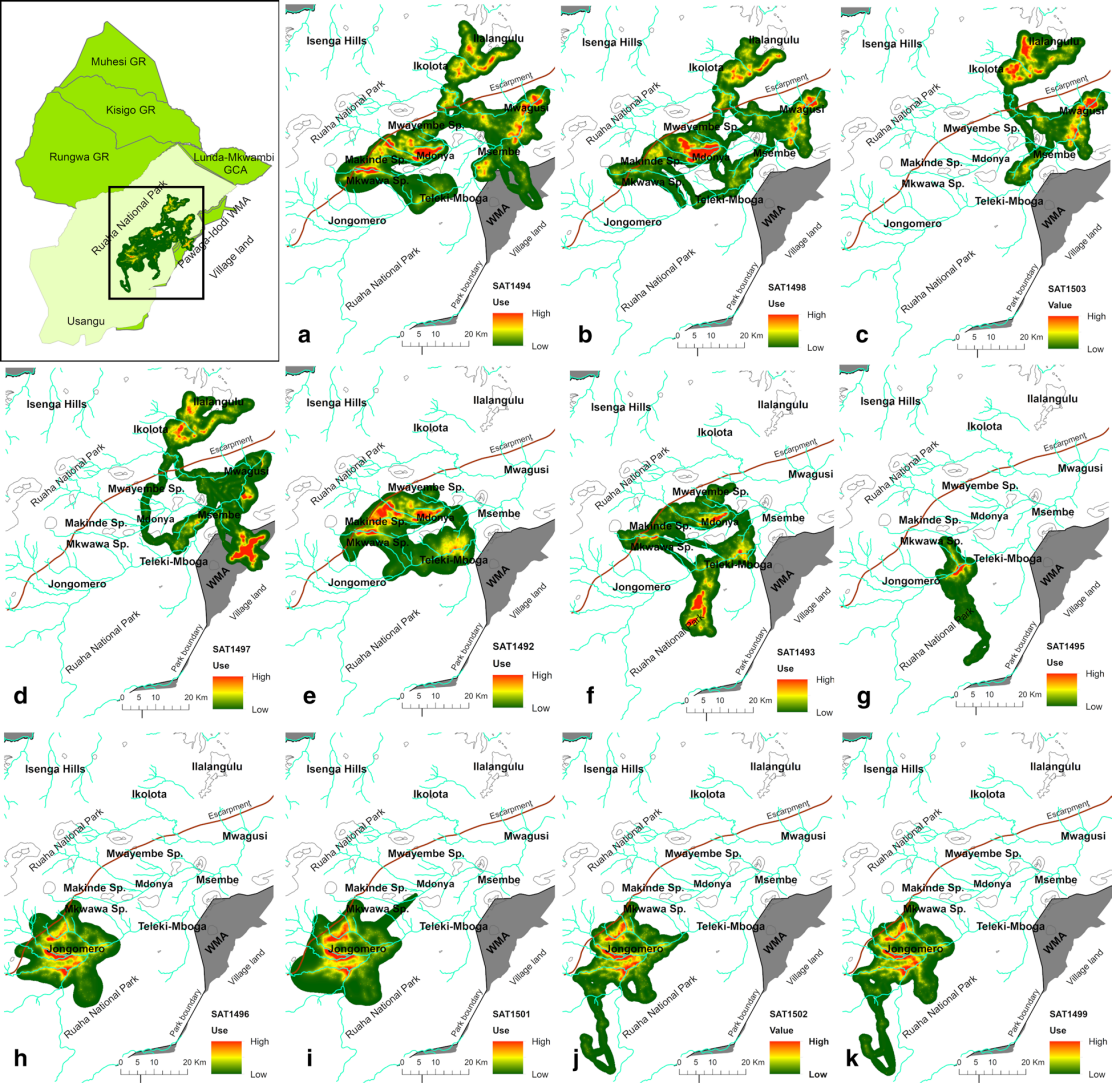
Brownian bridge models for 11 adult female African buffaloes in Ruaha National Park, Tanzania. The panels show the individual models for buffaloes collared near Mwagusi (**a**–**d**), TelekiMboga (**e**–**f**), TelekiMboga/Jongomero (**g**), and Jongomero (**h**–**k**). The collars were active between October 2014 and April 2017 (see Table 1 for details on collar duration) (Creator and copyright holder: A. Roug)

A total of 5 adult cows were collared in a 700–1000 animal mixed (both males and females) buffalo herd near Mwagusi, 4 adult cows were collared in a large 400 + mixed buffalo herd near Jongomero, 2 adult cows in a mixed 100–200 animal large herd near TelekiMboga, and one in a small, 30–40 animal herd with mainly bulls and a few cows between TelekiMboga and Jongomero (Figs. 1, 2). Ten of the buffaloes were collared in October 2014 and two in September 2015. One collar from 2015 was only active for approximately 4 months (SAT1500), and data from this animal were not included in the habitat use, home range analyses, or movement analyses.

### Home ranges and movement

The 95% and 50% home range sizes, as estimated by computing isopleths of the utilization distribution using Brownian Bridge Models (BBMM), were calculated for each individual buffalo within four time periods (November–May = wet season, and June–October = dry season for 2 years) as well as overall (Table 1). The areas that the buffaloes utilized varied with the largest for SAT 1494 (overall 95% estimated home range = 601 km^2^) and smallest for SAT 1495 (overall 95% estimated home range = 73 km^2^). Estimated home ranges were larger in the wet season compared to the dry season for all the collared buffaloes, and buffaloes consistently stayed closer to permanent water sources in the dry season.

**Table 1:** Size (in km^2^) of the 95% and 50% isopleth of the utilization distribution estimated using Brownian Bridge Models (as an approximation of home range sizes), of each collared buffalo in Ruaha National Park, Tanzania in the rainy (November–May) and dry (June–October) seasons as well as overall (entire time period)

Location collared		MWG	MWG	MWG	MWG	TKM	TKM	TKM/JGM	JGM	JGM	JGM	JGM
Time period	% use	SAT1497	SAT1498	SAT1503	SAT1494	SAT1492	SAT1493	SAT1495	SAT1496	SAT1499	SAT1501	SAT1502
Oct14–May15	95	219	306	290	254	–	205	86	215	246	174	253
Oct14–May15	50	47	59	67	55	–	30	12	44	46	39	49
Jun15–Oct15	95	129	137	136	142	–	98	9	109	100	112	98
Jun15–Oct15	50	27	29	27^a^	28	–	35	1	28	23	28	23
Nov15–May16	95	84	183	–	256	203	101	50	247	203	254	183
Nov15–May16	50	15	25	–	38	42	13	10^a^	57	44	60	42
Jun16–Oct16	95	80	35	–	149	139	51	–	109	96		100
Jun16–Oct16	50	16^a^	6^a^	–	21^a^	27	8^b^	–	27^a^	23^a^		24^a^
Nov16–Apr17	95	–	–	–	–	345	–	–	–	–	–	–
Nov16–Apr17	50	–	–	–	–	67^a^	–	–	–	–	–	–
Entire time period	95	357	488	290	601	296	304	73	289	283	297	283
Entire time period	50	67	88	67	123	68	47	7	63	58	63	58
Month ended^a^		Sept 16	Aug 16	Oct 15	Oct 16	Apr 17	Nov 16	Jan 16	Oct 16	Sept 16	May 16	Oct 16

The space use by year is shown in order to compare variations between seasons and years. MWG = Mwagusi, TKM = TelekiMboga, JGM = Jongomero

(–) no data

^a^Month ended: The last month that the collar was emitting points. For example, the collar SAT 1497 ceased working in September 2016

^b^For SAT 1493, the data from November 2016 is included in the “entire time period” but not in the Jun 16–Oct 16 time window

Early in the 2014–2015 wet season, the buffaloes collared in the same herd near Mwagusi (SAT 1494, 1497, 1498, and 1503, Fig. 2a–d) split up into separate groups as reflected of moving several kilometers apart from each other, but then reunited in the middle of the wet season and migrated to the high elevation plateau near Ilangulu (Fig. 2a–d). In the second wet season, the Mwagusi buffaloes split again without reuniting, with SAT 1494 (Fig. 2a) and SAT 1498 (Fig. 2b) moving to the locations near Mdonya, and SAT 1497 (Fig. 2d) moving into the wildlife management area between the park and village land. In contrast, the movement of the Jongomero buffaloes (Fig. 2h–k) was quite consistent across years, with buffaloes moving closer to the Great Ruaha River in the dry season and into more rugged and remote areas near the escarpment in the wet season. No spatial overlap between the Jongomero buffaloes and other buffaloes were observed (Fig. 2). The buffaloes collared near TelekiMboga (SAT 1492 and 1493, Fig. 2e–f) spent the first wet season near Mdonya, but in the subsequent wet season, SAT 1492 (Fig. 2e) appeared to switch herds and join the Mwagusi buffaloes (SAT 1494 and 1498, Fig. 2a, b) near Mdonya, whereas SAT1493 (Fig. 2f) moved across the Ruaha River and spent the entire wet season south of the Great Ruaha River. In the following dry season both buffaloes stayed near the river around TelekiMboga (Fig. 2e–f).

**Fig. 3:**
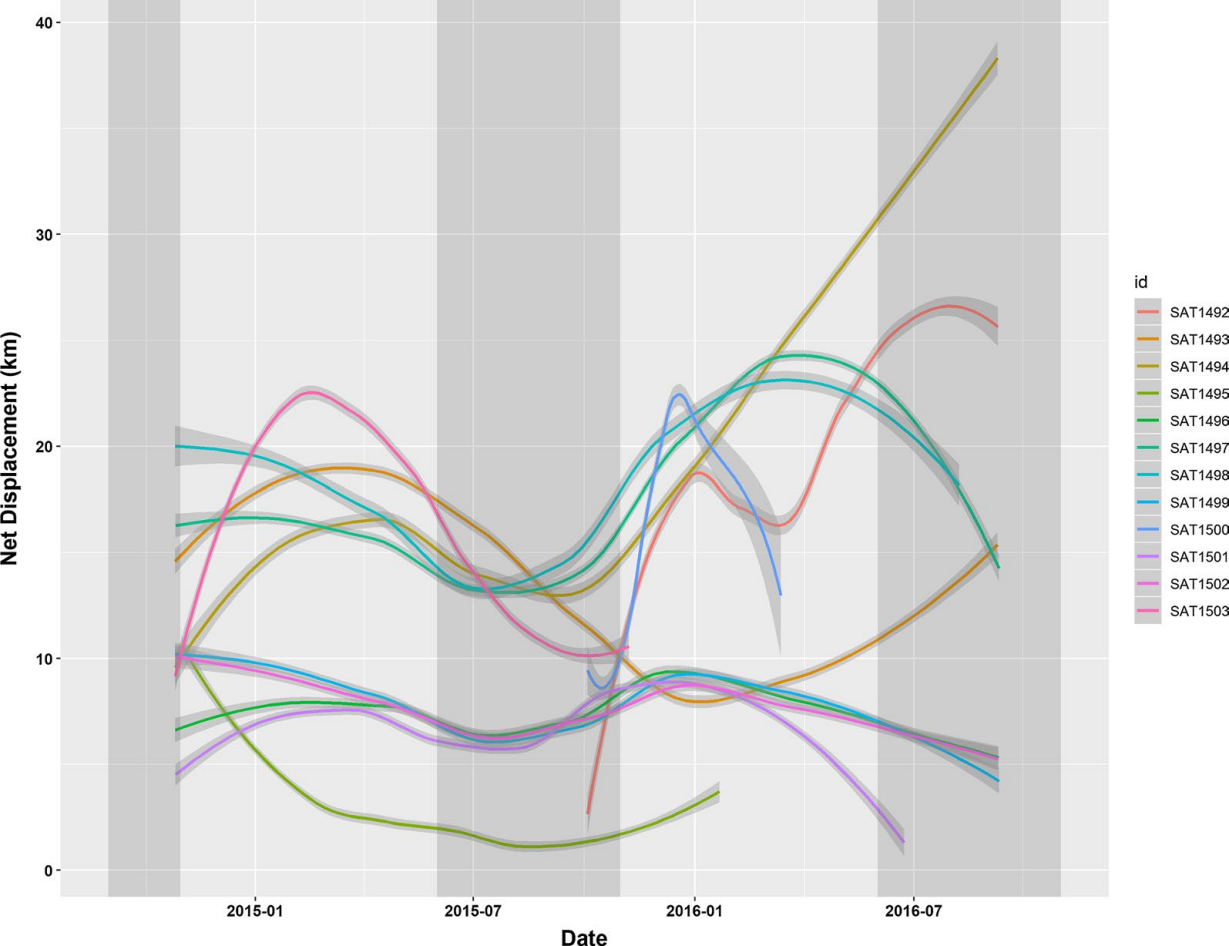
Net displacement in kilometers from location of collaring by year for African buffaloes in Ruaha National park between November 2014 and October 2016. The dry season is indicated with darker shading and wet season with lighter shading. In the first year, buffaloes largely returned to the previous dry season’s habitat near the Ruaha River as illustrated by the distances from the collaring location approaching zero in September–October 2015. In the second year, 3 animals (SAT 1493, 1494, and 1492) did not return to the same location after the second wet season

These movements were reflected as net displacement from the location of capture (Fig. 3). All buffaloes were collared during the height of the dry season, and then showed movements away from the Great Ruaha River in the wet season for all but one animal (SAT1495), which appeared to be resident (Fig. 3). The distances traveled were largest for the buffaloes collared near Mwagusi (SAT1494, 1497, 1498, and 1503, Figs. 2a–d, 3), followed by the two buffaloes collared near TelekiMboga/Mdonya (SAT1492 and 1493, Figs. 2f, g, 3). The Jongomero buffaloes (SAT 1496, 1499, 1501, and 1502, Figs. 2h–k, 3) only moved a short distance from the dry season habitat. After the wet season, the buffaloes largely returned to the previous dry season’s habitat by July, except for in the second wet season where two buffaloes collared near Mwagusi (SAT1494 and 1492, Fig. 2d, e), who had moved to Mdonya during the second wet season, did not return to the previous dry season’s habitat.

### Resource selection

**Fig. 4:**
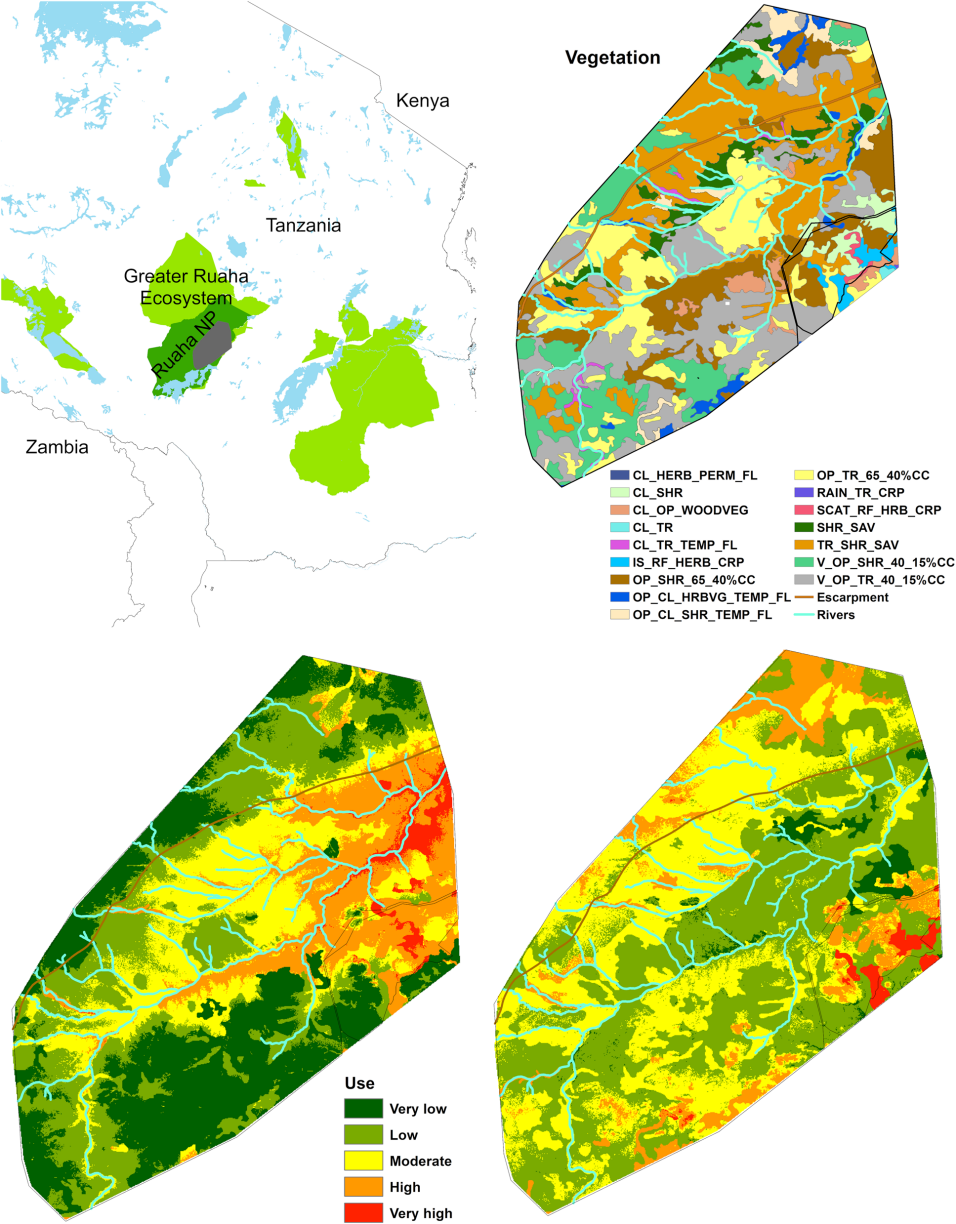
Vegetation and habitat selection in June–October (dry season) and November–May (wet season) within a polygon surrounding all collar points from 11 adult female African buffaloes collared in Ruaha National Park between October 2014 and April 2017 (see Table 1). The relative probability of use (Use) was based on the habitat selection models shown in Table 2. Abbreviations for vegetation types: CL_HERB_PERM_FL = closed herbaceous vegetation on permanently flooded land, CL_SHR = closed shrubs, CL_OP_WOODVEG = closed to open woody vegetation (thicket), CL_TR = Closed trees, CL_TR_TEMP_FL = closed trees on temporarily flooded land, IS_RF_HERB_CRP = isolated (in natural vegetation or other) rainfed herbaceous crops, OP_SHR_40_65% CC = open shrubs with 40–65% crown cover, OP_CL_HERBVEG_TEMP_FL = open to closed herbaceous vegetation on temporarily flooded land, OP_CL_SHR_TEMP_FL = open to closed shrubs on temporarily flooded land, OP_TR_40_65% CC = open trees with 40–65% crown cover, RAIN_TR_CRP = rainfed tree crop (mixed unit with natural vegetation or other), SCAT_RF_HERB_CRP = scattered rainfed herbaceous crop, SHR_SAV = shrub savannah, TR_SHR_SAV = trees and shrub savannah, V_OP_SHR_40_15% CC = very open shrubs with 15–40% crown cover, V_OP_TR_15_40% CC = very open trees with 15–40% crown cover (Creator and copyright holder: A. Roug)

Our top models for both the dry season and wet season contained most of the AIC weight (wi > 0.65 for the dry season and 0.42 for the wet season). Models ranked below the top model within each season were judged to contain uninformative parameters or coefficients [18] that were so similar to the top model for each season that we elected not to model average. The top model for dry season versus wet season were similar with only slope missing from the dry season and aspect excluded from the wet season. In the dry season, the buffaloes selected habitats at lower elevation and in more rugged terrain closer to the river compared to the wet season (Table 2, Fig. 4). All vegetation types except closed to open woody vegetation were preferred in comparison to the reference category (very open trees with 15–40% crown cover), with the strongest selection for open to closed herbaceous vegetation on temporary flooded land, closed shrubs, open shrubs or with 40–65% crown cover, closed trees on temporarily flooded land, isolated rainfed herbaceous crops, and scattered rainfed herbaceous crops (Table 2, Fig. 4). The latter two vegetation types were only present in the wildlife management area adjacent to the park, where SAT1497 spent part of a dry season and an entire wet season (Fig. 2d). In the wet season (November–May), the association with distance to the river was weaker than in the dry season, and the selection for vegetation types containing more shrub and trees was stronger, such as closed to open woody vegetation, shrub savannah, open to closed shrubs on temporarily flooded land, and closed trees on temporarily flooded land (Table 2, Fig. 4).

**Table 2:** Dry season (June-October) and wet season (November–May) resource selection models for 11 adult female African buffaloes in Ruaha National Park, Tanzania

Parameter	June–October		November–May	
	Coefficient	SE	Coefficient	SE
Intercept	− 0.076	0.192	− 0.762	0.069
ELEV (m)	− 0.761	0.228	0.261	0.013
Rugged	0.076	0.016	− 0.024	0.013
DISTRIV (m)	− 0.126	0.021	− 0.067	0.015
Aspect	− 0.045	0.014	–	–
Slope (%)	–	–	− 0.055	0.012
V_OP_TR_15_40% CC	Ref	Ref	Ref	Ref
OP_TR_40_65%CC	0.610	0.057	0.464	0.042
TR_SHR_SAV	0.603	0.055	0.843	0.041
OP_SHR_40_65% CC	1.141	0.056	0.809	0.041
CL_OP_WOODVEG	− 1.505	0.525	0.485	0.179
SHR_SAV	0.412	0.069	1.276	0.047
OP_CL_HRBVG_TEMP_FL	2.007	0.074	1.407	0.057
V_OP_SHR_15_40% CC	0.677	0.099	0.277	0.059
OP_CL_SHR_TEMP_FL	0.399	0.093	1.602	0.056
CL_TR_TEMP_FL	0.923	0.121	1.079	0.098
CL_SHR	1.506	0.181	1.704	0.135
IS_RF_HERB_CRP	2.125	0.239	2.893	0.157
SCAT_RF_HRB_CRP	2.362	0.203	2.594	0.176
CL_TR	–	–	10.064	43.955

The collars were active for varying durations between October 2014 and April 2017. Models were generated using mixed-effects logistic regression models with a random intercept specified for each buffalo and herd. The continuous covariates were standardized to improve model performance and allow for comparison of effect sizes across variables. A priori models were developed for each season and then ranked by model weight using the Akaike Information Criterion. ELEV (m) = elevation in meters, RUGGED = ruggedness, DISTRIV (m) = distance to nearest river in meters, V_OP_TR_15_40% CC = very open trees with 15–40% crown cover, OP_TR_40_65% CC = open trees with 40–65% crown cover, TR_SHR_SAV = trees and shrub savannah, OP_SHR_40_65% CC = open shrubs with 40–65% crown cover, CL_OP_WOODVEG = closed to open woody vegetation (thicket), SHR_SAV = shrub savannah, OP_CL_HERBVEG_TEMP_FL = open to closed herbaceous vegetation on temporarily flooded land, V_OP_SHR_15_40% CC = very open shrubs with 15–40% crown cover, OP_CL_SHR_TEMP_FL = open to closed shrubs on temporarily flooded land, CL_TR_TEMP_FL = closed trees on temporarily flooded land, CL_SHR = closed shrubs, IS_RF_HERB_CRP = isolated (in natural vegetation or other) rainfed herbaceous crops, SCAT_RF_HERB_CRP = scattered rainfed herbaceous crop, CL_TR = closed trees, SE = standard error, Ref = reference category for categorical variables

### Daily movements

**Fig. 5:**
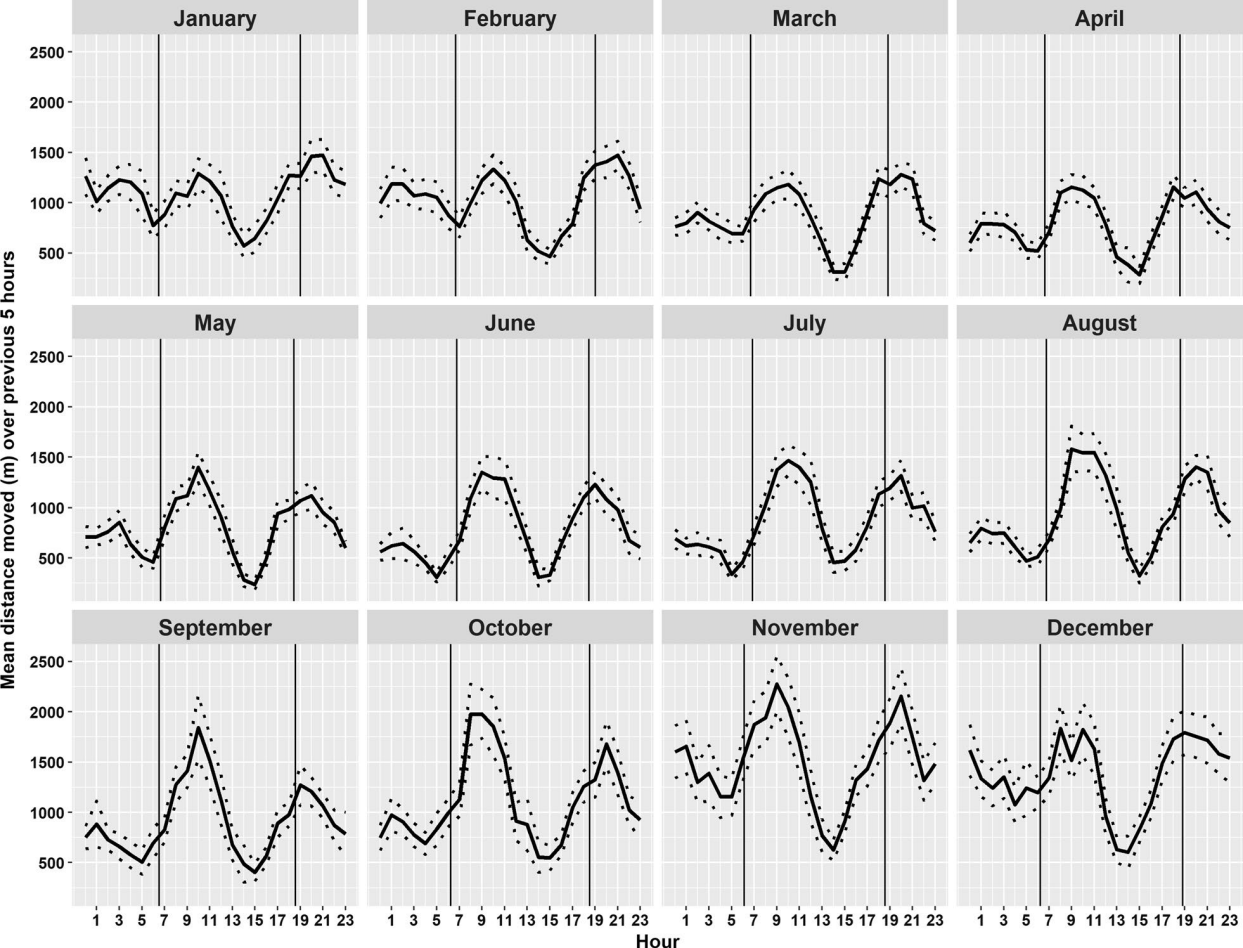
Mean and 95% confidence intervals of distance moved (in meters) in the previous 5 h by time of day and month, based on data from 11 collared adult female African buffaloes from 4 herds in Ruaha National Park, Tanzania. The time of sunrise and sunset is indicated for each month by the black vertical lines

The distance traveled in the previous 5 h was highest for points collected at 10.00 h (mean over all data = 1505 m, SD = 1107 m, n = 1245 points) and 20.00 h (mean over all data = 1430 m, SD = 969 m, n = 1247 points), and lowest at 14.00 h (mean = 471 m, SD = 627 m, n = 1235 points), indicating that the buffaloes were crepuscular with the highest activity levels in the late morning and evening and lowest activity level in the heat of the day (Fig. 5). The daily movements increased with progression of the dry season and peaked in November, where the average distance traveled in the last five hours was over 2000 m twice a day (Fig. 5). The shortest average daily distances traveled over the previous 5 h were observed in April, where the mean peak movements in the previous 5 h where less than 1250 m twice a day (Fig. 5).

The average total distance moved in 24 h was 4.9 km (Standard deviation (SD) = 2.9 km) in November–May and 4.2 km (SD = 1.9 km) in June-October. The longest distances were traveled during November at the end of the dry season and beginning of the wet season (mean daily distance = 6.9 km, SD = 3.6 km). The shortest daily distances traveled occurred in the wet season in April–June (mean 3.6 km, SD = 1.6–1.8 km). Across both seasons combined, the average daily distance traveled was 4.6 km (SD = 2.6 km, n = 6486).

## Discussion

Our study showed that the space use (estimated home ranges) of the individual collared buffaloes varied widely and were consistently larger in the wet season than in the dry season. Buffaloes showed strong preference for habitats near the Great Ruaha River in the dry season and less association to permanent water sources in the wet season. With the exception of one buffalo, all collared animals completed a wet season migration of varying distances, and daily distances traveled were longest during the late dry season and shortest in the height of the wet season. Several buffaloes appeared to switch herd during the study period. Buffaloes utilized the Wildlife Management Area on the southeastern border of the park, but did not venture into game reserves bordering Ruaha to the north and northeast.

### Home ranges and movements

During the dry season in Ruaha, water is limited to a few springs and the Great Ruaha River, constraining the buffaloes to stay much closer to the river than in the wet season, when seasonal pools and springs make the animals much more independent of the permanent water sources. Consequently, the area utilized by the buffaloes were generally larger in the wet season than in the dry season and the buffaloes showed a stronger selection for shrubby and woody habitats in the wet season (Table 2, Fig. 4). Shorter daily travel distances for collared buffaloes in the middle of the wet season, especially during April–May, compared to the beginning of the wet season (November–December) can be explained by the fact that some animals ranged widely in the beginning of the wet season before settling into an area with abundant forage for the wettest month of the year (Fig. 5). The size of the areas utilized were quite variable among the individual animals, and the largest area was observed with SAT1494 (95% estimated home range size of 601 km^2^ across all season) and the smallest with SAT1495 (95% estimated home range size of 73 km^2^ across all seasons). SAT1494 was collared in a 700 + animal herd near Mwagusi, whereas the herd with SAT1495 likely did not have more than 20 animals at any time. Larger home range sizes for buffaloes belonging to larger herds has been reported in buffalo herds from the Kruger National Park in South Africa and the Caprivi strip in Namibia [7, 19]; however, whether the estimated home range sizes truly were representative of what the buffaloes do every year is difficult to ascertain based on only 2 years of data. The 2015–2016 wet season had unusually high levels of precipitation [20] and water was likely not a limiting factor anywhere in the park. This may have influenced how buffaloes moved in the second year. In comparison, home ranges from adult female African buffalo collared near the Caprivi strip in Namibia ranged from 5.5 to 564.7 km^2^ using the 90% Local Convex Hull method [7]. In the Klaserie Private Nature Reserve in South Africa, the estimated home range sizes ranged from 170.7 to 327 km^2^ using the same methodology [4], and home ranges for two breeding herds in the Sengwa Wildlife Research Area of Zimbabwe were reported as 207 and 286 km^2^ [21].

Another factor that may have influenced the home range size measured in several buffalo was herd switching, or, for SAT1494 and 1498, at least movement into areas that was occupied by different buffaloes during the previous wet season (Fig. 2a, b). Herd switching was also reported from Botswana buffalo herds, where 7 out of 45 collared adult female buffaloes switched herds [22], but contradict earlier literature that generally considered buffalo cows to inhabit stable herds without inter-herd movements [9, 23]. The difference between the earlier literature and the observations in our and the Botswana study can likely be explained by the use of GPS collars in the Botswana and our study, as more fine scale movement data can be obtained with GPS technology.

While herd switching was observed, herds also appeared to avoid each other to some degree. Two buffaloes were collared near TelekiMboga (SAT1492 and 1493, Fig. 2e, f), and while SAT1492 joined the buffaloes coming from Mwagusi (SAT 1494 and 1498, Fig. 2a, b) in the second wet season (Fig. 2e), SAT 1493 moved south of the river and did not appear to share the same area as SAT1493, 1494, and 1498 in the subsequent dry season (Fig. 2f). Also, the Jongomero buffaloes (Fig. 2h–k) never directly overlapped with areas occupied by any of the other collared buffaloes even though they frequently moved into areas adjacent to the range occupied by SAT1495 (Fig. 2g). One collared buffalo (SAT 1497) spent an entire wet season in the wildlife management area outside the park (Fig. 2d). It is possible that the buffaloes that ventured into the wildlife management area were trapped there during the wettest time of the year as the river was unusually high during the second wet season and likely did not allow for buffaloes crossing safely, especially with small calves. More data are needed in order to understand the observed movements and elicit any consistent patterns in the Mwagusi and TelekiMboga herds.

Movement of animals can be classified as migratory, mixed migratory, dispersal, or non-migratory using net square displacement [24, 25]. From a buffalo study in the Caprivi strip of Namibia a fifth class of migratory behavior, “expanders”, has been suggested, which are animals that expanded their range rather than moving entirely away from their dry season home ranges during the wet season [8]. The Jongomero buffaloes did move from their dry season location to areas closer to the escarpment, but also regularly returned to areas that were used during the dry season, and could, therefore, tentatively be classified as expanders. The buffaloes collared near Mwagusi and TelekiMboga/Mdonya behaved differently in the 2 years they were observed and, based on available data, could therefore be categorized as mixed migratory, and SAT1495 did not migrate (Fig. 2g) and could therefore be classified as non-migratory or resident [8].

### Daily movements

The patterns of daily distances traveled were consistent with field observations, as buffaloes usually were seen arriving at the river between 8 and 11 in the morning, and again around 16 and 19 in the afternoon and evening (Fig. 5). The daily movements increased with progression of the dry season and peaked in November, which is the last month of the dry season, when animals are forced to travel longer distances to find adequate forage but have to return to the river on a daily basis to drink. The end of November is also the beginning of the rainy season when buffaloes moved away from the area around the Great Ruaha river to their wet season habitats. The shortest daily distances traveled were observed in April (Fig. 5) which is the middle of the wet season, where abundant forage and availability of seasonal pools makes it unnecessary for the buffaloes to travel long distances to find water.

The average daily distance moved of 4.6 km (SD = 2.6 km) is lower than what has been reported in other buffalo herds. Buffalo herds in Cameroon, for example, moved an average of 7.2 ± 2.62 km in the dry season and 5.6 ± 0.87 km in the wet season [26]. Buffaloes in the Sengwa Wildlife Research Area of Zimbabwe moved an average of 6.1 km (SD = 2.02 km) in the dry season [21], and buffaloes in Rwenzori National Park in Uganda moved an average of 9.6 km per day (range = 5.2–14.4 km) [27]. In contrast, breeding herds of buffaloes in Kruger National Park moved an average of only 3.35 km per 24 h (standard error = 0.35 km); however, this distance did not vary with season, indicating that reliable water and grazing was available in both the wet and dry seasons [28].

### Resource selection

As expected, buffaloes selected habitats near the river in the dry season compared to the wet season when rainfall made water abundant across the park. Similar observations have been reported in other locations; e.g. in the Caprivi strip of Namibia buffaloes moved to the flood plain near the rivers and adjacent woodland in the dry season and moved away from rivers into areas with ephemeral water in distant woodland in the wet season [7]. However, in studies from the Doornkloof Nature Reserve in the Nama-Karoo in the Northern Cape Province of South Africa, and Klaserie Private Nature Reserve in South Africa, buffaloes ranged farther and wider in the dry season than in the wet season in order to find adequate forage [4, 29]. Similar observations were made in Kruger National Park, South Africa, where buffaloes ranged farther in dry years than in wet years [12].

None of the collared buffaloes ventured into the northern game reserves during the hunting season, and only one herd spent significant time outside the park within the wildlife management area. Harvest of animals is therefore unlikely to have a direct population impact for the observed herds. Whether other herds in which no collars were placed are impacted by hunting pressure cannot be elicited from our data.

Nonetheless, buffaloes are occasionally detected with camera traps on village land (Ruaha Carnivore Project, personal communication), indicating that they do spend time outside the park. The extent of contact with livestock and consequent risk of disease transmission between these species is unknown. Studies from Uganda and Zimbabwe showed that direct contact between cattle and buffaloes is unlikely [30, 31] and disease transmission between buffaloes and cattle therefore likely occurs through shared environments and not through direct contact.

### Conclusions

The seasonal pattern of buffalo movement observed suggests that the Ruaha buffaloes may be restricted in their movement during the dry season due to dependence on a few perennially available water sources, which consequently increases the pressure on the habitat around these areas. Hunting is unlikely to have major impact on the buffaloes in the core herds of the park, and buffaloes generally appeared to be in good health with adequate calf recruitment as observed during the capture work and based on demographic surveys, although the recruitment varied with rainfall [32]. Our study findings may therefore indicate that the buffalo population in Ruaha declined due to seasonal reductions in the flow of the areas main water source, the Great Ruaha River. As a water-dependent species, the area of suitable and reachable habitat would have diminished drastically with dry season cessation of water flow. Additional movement data, including collaring of buffaloes belonging to herds along the border of Ruaha’s protected areas, is needed to fully delineate the home ranges and habitat preferences. Long term monitoring of water flow and buffalo populations trends may increase the understanding of the impact of seasonal water scarcity on Ruaha’s wildlife, and benefit the conservation of buffaloes in Ruaha National Park. On a broader scale, our observations demonstrate the importance of linking population data, migration, habitat preferences, and ecosystem changes in order to understand population dynamics of large ungulate species in Africa and beyond.

## Methods

### Study area

Ruaha National Park, Tanzania’s largest national park, is a part of the Rungwa-Kizigo-Muhesi ecosystem and covers an area of 20,226 km^2^ [33]. The park is bordered by game reserves to the north-east and a wildlife management area to the south-east (Fig. 1). Together, this ecosystem spans an area of over 45,000 km^2^, making it one of the largest contiguous wilderness areas in the world [13]. The rainy seasons extend from November to February and from March to April, and annual mean precipitation is 500–800 mm [33, 34]. The vegetation is dominated by miombo woodland in the south-western part of the park and commiphora-combretum woodland and acacia Savannah in the central and eastern parts of the park [34]. Main rivers include the Great Ruaha, Mzombe, Mdonya, Mwagusi and Jongomero rivers [33]. The southern portion of the park is located within a valley, and the valley edge creates a steep escarpment extending from the north-east to the south-west [33] (Fig. 1).

### Collaring

The locations of major buffalo herds within Ruaha National Park were known from annual demographic surveys [32] as well as from local tour guides and park rangers. During September–October of 2014–2015, a total of 12 adult buffalo cows from 5 herds were immobilized via dart delivered from vehicles using 8–10 mg of etorphine hydrochloride (M99, 9.8 mg/ml, all drugs obtained through Alphavet, Arusha, Tanzania) and 60–100 mg of azaperone (100 mg/ml). Immobilization was reversed with 36 mg diprenorphine (M5050, 12 mg/ml) and 80 mg naltrexone (50 mg/ml) injected via hand-syringe intravenously. All 12 adult cows were fitted with iridium satellite GPS collars (African Wildlife Tracking, Pretoria, South Africa, weight 1.7 kg, length of belt 1060 cm). The collars were programmed to transmit 5 points per day by satellite uplink until September 2016, where after the transmission was slowed to 2 times per day with 12 and 13 h between each uplink. The frequency of transmission was slowed in the hope of being able to obtain a third year of wet season data; however, for all but one animal, the batteries failed before the third wet season.

### Determination of space use

The probability of space use was estimated for individual buffaloes using Brownian Bridge Models (BBMM) using the packages BBMM in R [35]. Rasters and shapefiles of the space use, as an estimate of the home ranges, were created with the R-packages rgdal, maptools, and raster [35]. The 99, 95, and 50% isopleth of the utilization distribution were calculated in square kilometers for each individual buffalo by season and year in order to compare space use by season and variation between years.

### Resource selection

We used a resource selection function (RSF) to evaluate patterns of habitat selection of buffaloes in RNP. Vegetation and river data were obtained from Ruaha National Park, and topography data were obtained using the Advanced Spaceborne Thermal Emission and Reflection Radiometer (ASTER) Global Digital Elevation Model (GDEM) [36]. The elevation (in meters), slope (percent), aspect (north, south, east, west), distance to nearest river (Euclidian distance, in meters), and ruggedness were calculated in ArcMap (vs. 10.6, ESRI, Redding, CA, USA) from the GDEM data. We evaluated habitat selection at the 3rd order [37]. For each buffalo and season (November–May = wet season, and June–October = dry season), data on vegetation type, elevation, slope, ruggedness, aspect, and distance to river were extracted for each collar point. Shapefiles delineating the 99% isopleth of the utilization distribution generated with the Brownian bridge models were used as boundaries for creating an equal number of random points as there were collar points. Resource selection was evaluated within a used-available design at the individual animal level [37, 38]. We used mixed-effects logistic regression models with a random intercept specified for each buffalo and herd (3rd order selection) using the glmer function (nAGQ optimization algorithm) within package lme4 in the software R [39]. We also standardized the continuous covariates (z-score) to improve model performance and allow for comparison of effect sizes across variables. A priori models were developed for each season and then ranked by model weight (*w*_*i*_) using the Akaike Information Criterion (AIC) [40]. We carefully inspected model output to avoid use of models with uninformative parameters [18].

Coefficients from models of habitat selection were then used to generate a map of the relative probability of use across our study area. This model was projected at 30 m spatial resolution within a minimum convex polygon surrounding all buffalo points. Data on elevation, slope, aspect, ruggedness, distance to river, and habitat type were extracted for each point, and the averaged regression equation was applied to each point in order to generate the relative probability of use [p = (e^model^/1 + e^model^)]. The probabilities of use were then mapped in ArcMap for the wet season and dry season.

### Daily movements

The distribution of the distance (in meters) moved between each 5-hour collar transmission was shown using the mean and 95% confidence intervals by hour of the day and month for all buffaloes combined using the package ggplot2 [41]. The net displacement from the location of collaring was determined using established methods [24] with the packages adehabitatLT [42] and dplyr [43] in R, and plots were generated using the package ggplot2. Only the data from when the collars were transmitting every 5 h were included in this analysis.

## Data Availability

The data is available from the corresponding author upon reasonable request and with permission from Tanzania National Parks.

## Abbreviations

HALI: Health for Animals and Livelihood Improvement Project
MWG: Mwagusi
TKM: TelekiMboga
JGM: Jongomero
ELEV (M): elevation in meters
RUGGED: ruggedness
DISTRIV (M): distance to nearest river in meters
V_OP_TR_15_40% CC: very open trees with 15–40% crown cover
OP_TR_40_65% CC: open trees with 40–65% crown cover
TR_SHR_SAV: trees and shrub savannah
OP_SHR_40_65% CC: open shrubs with 40–65% crown cover
CL_OP_WOODVEG: closed to open woody vegetation (thicket)
SHR_SAV: shrub savannah
OP_CL_HERBVEG_TEMP_FL: open to closed herbaceous vegetation on temporarily flooded land
V_OP_SHR_15_40% CC: very open shrubs with 15–40% crown cover
OP_CL_SHR_TEMP_FL: open to closed shrubs on temporarily flooded land
CL_TR_TEMP_FL: closed trees on temporarily flooded land
CL_SHR: closed shrubs
IS_RF_HERB_CRP: isolated (in natural vegetation or other) rainfed herbaceous crops
SCAT_RF_HERB_CRP: scattered rainfed herbaceous crop
CL_TR: closed trees
SE: standard error
SD: standard deviation
ref: reference category for categorical variables in the regression model
hrs: hours
m: meters
BBMM: Brownian bridge models
AIC: Akaike Information Criterion
ASTER: Advanced Spaceborne Thermal Emission and Reflection Radiometer
GDEM: Global Digital Elevation Model
